# Cross-Domain Security Asset Management for Healthcare

**DOI:** 10.1007/978-3-030-69781-5_10

**Published:** 2021-01-28

**Authors:** Federico Stirano, Francesco Lubrano, Giacomo Vitali, Fabrizio Bertone, Giuseppe Varavallo, Paolo Petrucci

**Affiliations:** 8 Norwegian Computing Center, Oslo, Norway; 9 University of Trento and Fondazione Bruno Kessler, Trento, Italy; 10 Università degli Studi di Genova, Genoa, Italy; 11 IEIIT Institute, Consiglio Nazionale delle Ricerche (CNR), Genoa, Italy; 12 SINTEF A.S., Oslo, Norway; 13 Engineering Ingegneria Informatica S.p.A., Rome, Italy; 14 Instituto Superior de Engenharia do Porto, Porto, Portugal; 15 University of Padua, Padua, Italy; 16 LINKS Foundation, via Boggio 61, Turin, Italy; 17 ASL TO5, Moncalieri, Italy

**Keywords:** Healthcare, Critical infrastructure, Cross-domain security, Asset availability

## Abstract

Healthcare is one of the most peculiar between all Critical Infrastructures due to its context and role in the society. The characteristics of openness and pervasive usage of IT systems and connected devices make it particularly exposed to both physical threats, such as theft and unauthorized access to restricted areas, and cyber attacks, like the notorious wannacry ransomware that abruptly disrupted the British National Health System in May 2017. Even the recent COVID-19 pandemic period has been negatively characterized by an increase of both physical and cyber incidents that specifically targeted hospitals and undermined an essential public service like healthcare. Effective security solutions are necessary in order to protect and enhance the resiliency of the Critical Infrastructures. This paper presents the work being developed in the context of the SAFECARE H2020 project, that specifically considers the requirements for security of hospitals. A particular focus is given to the asset management that consider cross-domain aspects of security, like the physical location and virtual connections that link different components of a hospital. This allows advanced knowledge that enables to infer and forewarn of possible elaborated cyber-physical *kill chains*. This is particularly important and useful during crisis, as allows to have a holistic overview of the status of the hospital and the potential impacts of one or more incidents to the critical assets. The description and simulation of an attack scenario is also given, together with the description of the messages exchanged by the security systems and the information made available to security operators.

## Introduction

Hospitals, power supplies, water supplies, telecommunications and transports are just few examples of Critical Infrastructures (CIs). CIs provide vital functions to modern societies, and the protection of such infrastructures is a key issue at European level. Thus, the creation of systems able to provide threat prevention, detection, response, and, in case of failure, mitigation of impacts across infrastructures, populations, and environment, is now a primary concern.

Hospitals, and in particular typical European hospitals, are peculiar CIs, because of their context and role. Hospitals, in several cases, are placed in urban environments and sometimes in historical buildings. They are characterized by a considerable influx of various people (patients, visitors, medical staff, administrative staff, technicians) and the simultaneous presence of different organizations, such as universities.

Unlike public offices or other public buildings, hospitals have limited possibilities to restrict the access in one *single point of entry* and to create checkpoints and visitors control desks to check or store identity documents. Therefore, it is very challenging to manage and control the access to the hospitals public areas, making them particularly vulnerable to physical threats.

Besides people, that are the most critical asset, healthcare infrastructures are also characterized by the presence of a huge number of different technical equipment. Cryogenic system, RX systems, radioactive isotopes, big magnetic field systems, gases tanks, hyperbaric systems, picture archiving and communication system (PACS), laboratory information systems (LIS), are a small representation of the wide variety of critical assets that can be found inside hospital facilities.

Such components, as many others present in healthcare infrastructures, are becoming increasingly integrated and connected. Indeed, nowadays hospitals deeply rely on the IT network and IT systems. For example, the PACS and the LIS systems manage and exchange data through the hospital IT network. These systems allow the possibility to work with radiological images and laboratory tests. Attacks to such systems or the internal network of the hospital, could create several issues or seriously slow down the diagnosis process or patient treatment.

For example, a set of 19 vulnerabilities has been recently disclosed under the name of Ripple20 [1]. These vulnerabilities could allow the remote execution of code on IoT devices and embedded systems, posing a serious risk for patient’s health. It has been reported that the most affected sector is healthcare and more that 50000 medical devices such as infusion pumps have already been identified to be vulnerable [2].

Cyber-security solutions, implemented to protect these critical assets, provide a reasonable level of security, preventing such types of attacks and increasing the difficulty of reaching such systems from outside of the network.

While generally most of the cases of physical incidents are limited in scope, a physical intrusion like an access with tailgating or burglary or otherwise fraudulent could be preparatory for a following cyber attack, for example to exploit the mentioned vulnerabilities.

This combination of physical intrusion and the consequent cyberattack constitutes an example of a new category of threats that includes more complex but effective attacks. Thus, the border between physical and cyber domains is increasingly blurred. From the security point of view, an integrated approach that takes into account physical and cyber threats is then required.

During the recent COVID-19 pandemic, an increase of both cyber attacks (malware) and physical incidents (theft) has been observed. At the time of writing, SAFECARE tracked more than 150 incident reported in news from all over the world [3]. This is due to the opportunistic behaviour of criminals, that will try to take advantage of critical situations to gain profit.

This paper describes the integrated cyber-physical security solution being implemented in the context of the SAFECARE H2020 project^1^. A special focus is dedicated to the Hospital Availability Management System (HAMS), a sub-module of SAFECARE that provides a global overview of the hospital status and is integrated with the SAFECARE incident detection and impact evaluation functionalities. With its interfaces the HAMS shows what are the occurred incidents, what are their impacts and provides assets availability, updated when incidents occur.

## Related Work

Asset management, resources availability and bed occupancy are very important aspects to handle during the day-to-day operations of an hospital. This is even more critical during emergency situations and events like natural disaster, terrorist act or other hazards.

One of the most important example of software that is specifically designed for emergencies is SAHANA Disaster Management System (DMS) [4, 5]. SAHANA DMS was adopted in 2010 during the earthquake emergency in Haiti, in the city of Port-au-Prince. This system helped manage the flow of victims in Haiti, sharing data about hospital assets (department service, bed availability, staff availability) with emergency administrators.

Health Resources Availability Mapping System (HeRAMS) [6, 7] developed by the WHO and Global Health Cluster, is another important example. Its objective is to estimate the availability of services and resources in the hospitals placed in regions in crisis or health emergency. HeRAMS is based on questionnaires carried out in health care infrastructure to collect data concerning the availability of health resources and services such as health care personnel, beds, medical devices, and medicines. The questionnaires’ outcomes are published in an interactive dashboard to reflect the situation of health care resources.

The analysis of these systems showed that the data related to the availability of assets is updated manually (Sahana DMS); in the second case, the data of assets is updated using the results of questionnaires (HeRAMS).

More in general, a multitude of different solutions that handles various aspects of asset management (e.g. tracking of non-fixed assets, maintenance, inventory, bedding, software licensing, etc.) exists.

While the main objective of those systems is to enhance the efficiency of the hospital in normal conditions, the asset availability management solution described in this paper is specifically conceived to take into account the cyber and physical incidents detected by the platform and accordingly update the availability of impacted or potentially impacted assets.

## SAFECARE Architecture

The EU-funded SAFECARE project aims at developing an integrated solution to the cyber and physical threats which can affect healthcare infrastructures [8]. This is done through the implementation and deployment of multiple systems that recognize, correlate and analyze those threats and, when incidents occur, infer the impacts and start responses and damage mitigation mechanisms [9]. This will lead to an improvement in the security, protection and resilience of such services, allowing for a better response in case of external attacks specially during health related emergencies such as the current COVID-19 pandemic.

The SAFECARE architecture is composed of several modules, each with specific role, in this paper we will focus just on a subset of them that can be classified into two major functions:

- *Cyber and physical incident detection systems*
- *A centralised structure capable of storing and combining incoming data and of evaluating potential impacts when security incidents occur*

**Fig. 1.**
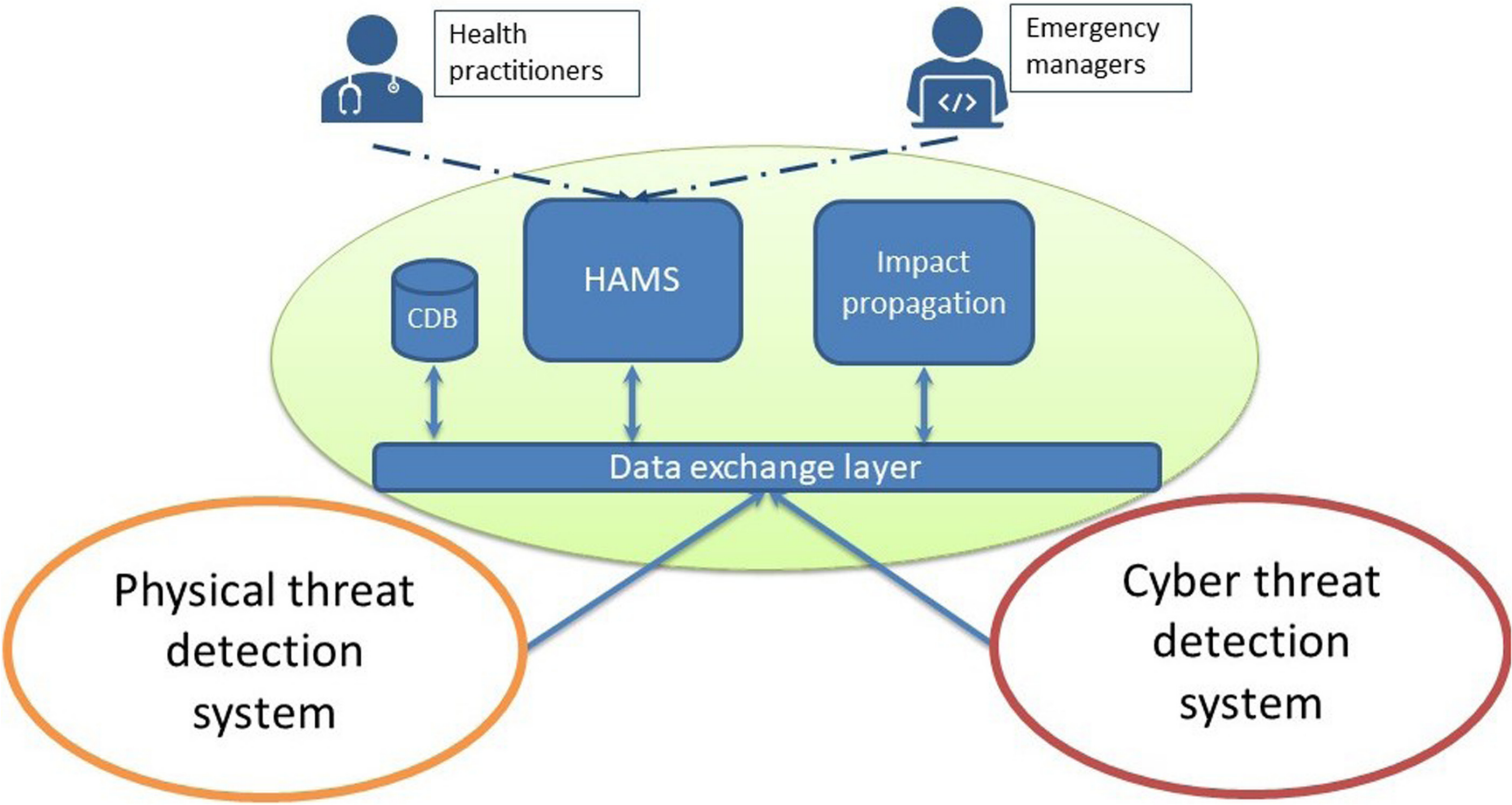
SAFECARE global architecture.

A simplified schema of the global structure is represented in Fig. 1. The connection between the different components is ensured by the Data Exchange Layer (DXL), which allows all the modules to communicate with each other in real time and provide relevant interfaces to exchange data.

### Threat Detection Systems

The Threat Detection Systems consists of physical and cyber security solutions implemented by smart modules and integrated technologies.

More specifically, physical security solutions embed intelligent video monitoring and interconnect building monitoring systems as well as management systems. The Building Threat Monitoring System (BTMS) module is the aggregation point for physical security and makes the link between physical systems and the rest of the architecture.

In the same way, the Cyber Threat Monitoring System (CTMS) connects the cyber systems to the rest of the architecture and is the central point for cyber security solutions, which consist of threat detection systems related to Information Technology (IT) and e-health systems.

Both physical and cyber security monitoring tools are interconnected thanks to the cyber-physical security solutions. These consist of intelligent modules whose role is to integrate different data sources and better take into account the combination of physical and cyber security threats.

### Central Database and Decision Modules

In order to cope with such a combined approach for the management of cyber and physical security, the SAFECARE project implements a Central Database (CDB): a single, unique repository that stores multiple types of data needed by the other modules in the platform.

In particular, it stores all the information related to assets, facilities, buildings, services inside the hospital, the relations among the assets (static data) and also the information that is generated by SAFECARE modules, such as incidents, impacts, the relation between them and all the other responses/messages (dynamic data).

The modules responsible for inferring cascading impacts of physical and/or cyber security incidents and providing information about health services availability are the Impact Propagation and Decision Support Model (IPDSM) [10] and the Hospital Availability Management System (HAMS) [11], respectively. In particular, the IPDSM’s role is to combine physical and cyber incidents that occur on assets, infer cascading effects as impacts that could potentially affect the same or related assets and, finally, alert other modules about the potential impacts and severity. The HAMS, on the other hand, elaborates the messages coming from the other modules to provide asset availability information.

### Incidents and Impacts Messages

The different modules communicate to each other by means of the MQTT protocol. In SAFECARE architecture, the broker is located in the DXL, while each module implements its own client. Messages are exchanged through a publish/subscribe mechanism, where clients publish on a specific topic and receive messages only on the topics they have previously subscribed over. Messages are encapsulated in the JSON format. While there are different kind of messages exchanged between SAFECARE modules, in this paper we focus on messages related to incidents and impacts.

An incident message is generated after a validation process carried out by a security operator, in order to verify that an event detected by the monitoring modules can be classified as an actual incident. The main fields contained in the incident message are: *incident identifier*, a unique reference in the whole system to a specific incident; *category*, the incident category that helps the analysis of impacts and availability; *severity*, that indicates how severe the incident is (the possible values are: *LOW*, *MINOR*, *MAJOR*, *CRITICAL*); *events*, a list of the events that led to the incident. Each *event* includes the list of the assets involved that may change their availability status, according to the procedure described in Sect. 4.

The impact message is composed by a field with the *incident identifier* of the related incident and a list of *assets* that are not directly involved by the incident but that may be impacted by its propagation. In fact, assets are associated to a set of parameters that express the likelihood that the asset can have consequences, according to the analysis performed by the IPDSM. One of these parameters is the *ImpactScore*, which gives a forecast of how much the asset is indirectly impacted by the related incident, 1 being totally unavailable and 0 not impacted at all, while intermediate values represent different degrees of impact.

Section 5.2 will provide an example of the incident and the impact messages generated by the sample scenario described.

## Hospital Availability Management System (HAMS)

This section describes the behavior of the Hospital Asset Availability Management system in response to events detected by the security systems.

### Evaluation of Impact and Asset Availability

When the HAMS receives an incident or an impact message, it must evaluate the content of message to verify whether the availability status of an asset needs to be modified or not. Asset availability status is represented with three parameters:

1. A Boolean value, stating if the asset is available or not.
2. A colour code (green, yellow, or red), acting like a traffic light. If the colour code is green, the asset is working normally. If the colour code is red, the asset has been heavily impacted by the incident and it is not available. If the colour code is yellow, the asset has been impacted by the incident, but it is still partially working and available, for example locally but not remotely.
3. A stability value (stable, improving, deteriorating), providing a simple description of the dynamic of status variations.

Upon arrival of an incident message, the system checks the incident category and couples it with the category of each related event, as not all the categories can provoke a change of availability in the assets. For each asset whose status needs to be changed, the severity parameter is checked to define if the new status colour for each asset will be *yellow* (in the case of *LOW* or *MINOR* severity) or *red* (in the case of *CRITICAL* or *MAJOR* severity). Any other variation in the availability of assets is computed after the reception of an impact message.

When an impact message is received, the *ImpactScore* value is checked to decide whether an asset availability status value needs to be updated or not. Using a threshold approach, an asset can be declared unavailable (with colour red if *ImpactScore* $\ge$ 0.8), partially available (available with colour yellow if 0.5 $\le$ *ImpactScore* < 0.8) or fully available (with colour code green if *ImpactScore* < 0.5) by the HAMS. The thresholds could be changed or customized depending on the asset category or the different requirements of each hospital.

### Asset-Centric Graph Visualization

The HAMS is implemented as a web application. Besides the server side, that connects this software with the SAFECARE system and implement all the logic to retrieve information from the database and receive all the useful messages, the HAMS implements a Graphic User Interface (GUI).

End-users involved in SAFECARE project provided their feedback about how to visualise asset affected by incident and taking into account the implicit hierarchical dependencies of assets in an hospital, an asset-centric graph visualisation has been developed.

**Fig. 2.**
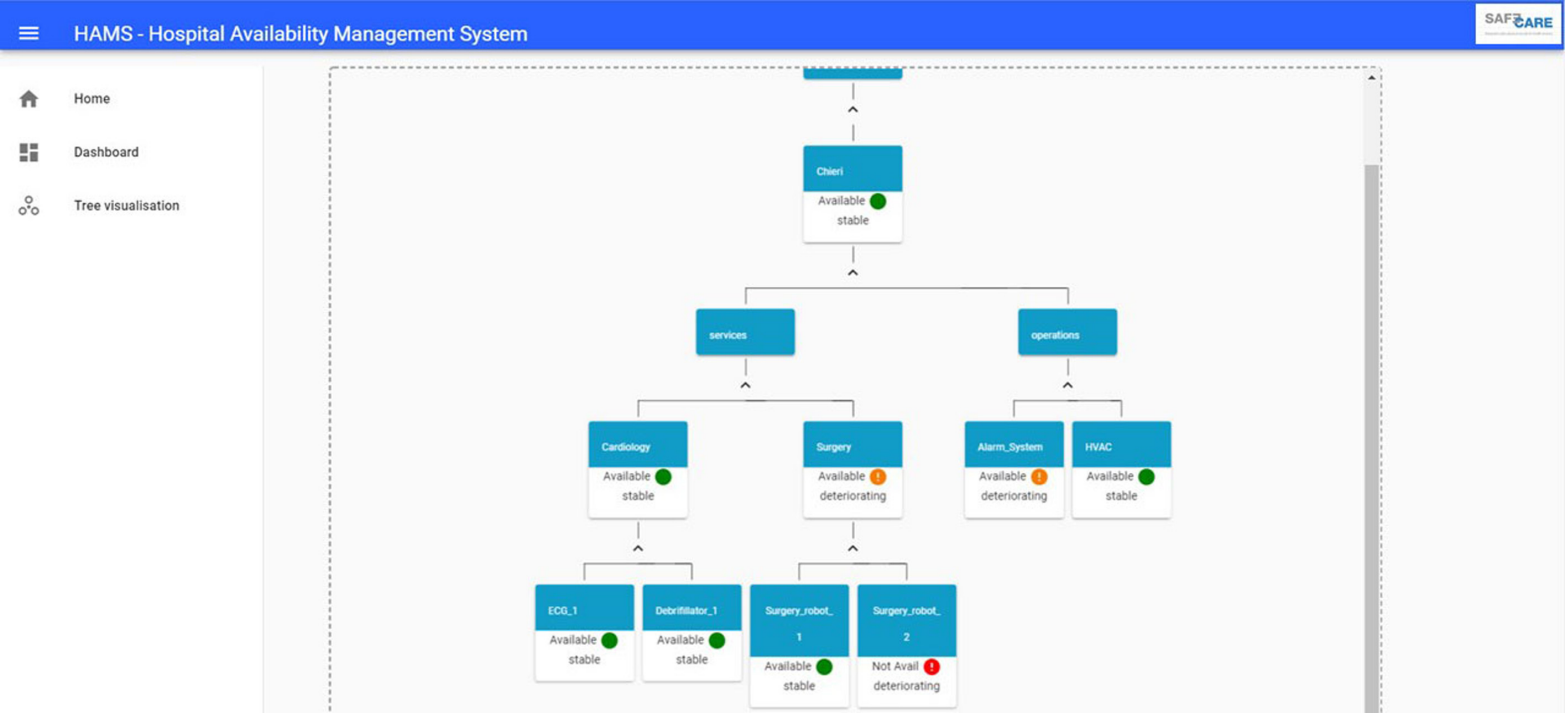
HAMS graph-based visualisation (Color figure online)

Figure 2 reports an example of this visualisation for a fictional hospital. Starting from a facility level, the graph provides information about the status of services and operations. In this terminology, services includes hospital departments and a list of medical devices belonging to each service. On the other side, there are operations, that include all the systems that are not related to the medical activities but that are essential for the correct operation of the hospital. Each node of the graph reports the status of the asset and in case an end node (an asset) is involved in an incident, user can visualise details about the incident or the incidents that caused the changes in the asset status.

### Interoperable Information Exchange

During the management of an emergency in the hospital sector, one of the main requirements is the capability to exchange the information in a timely manner, with a common data format for all the actors involved (hospitals, first responders, etc.). HAMS deeply relies on the EDXL-HAVE [12, 13] standard to represent data internally and to share them with other systems.

EDXL [14] is a set of standards released by OASIS to manage the entire emergency life cycle and provide a common framework to exchange and share information between different emergency systems. In particular, EDXL-HAVE (HAVE) is an XML messaging standard developed by OASIS in the context of emergency management.

A HAVE schema consists of a root element that uniquely identifies the organization that is responsible for the reporting facilities. Each facility is described through several attributes and a list of sub-elements that allow a complete description of hospital departments, services, and resources.

As the HAMS data model is based on the HAVE standard, the updated availability information can be easily exported in a XML message based on the HAVE schema. In this way, the output file can be exchanged with other hospitals or emergency management actors to better coordinate the operations in case of necessity.

## Sample Scenario

In order to design the modules and to test the developed solution, it is necessary to define plausible scenarios that combine physical and cyber attacks. Health organizations have a large variety of assets, which are essential for their operations. Therefore, it is necessary to firstly classify critical assets to have a global knowledge of the context. In the SAFECARE project, the following categories of assets that compose Healthcare organizations were identified:

- *Specialist personnel assets, (e.g., employees);*
- *Buildings and facilities, (e.g., power and gas supply, PLC room);*
- *Identification system, (e.g., badges, digital credentials);*
- *Networked medical devices, (e.g., medical devices connected to the internal data network);*
- *Networking equipment, (e.g., laptop, internet network);*
- *Interconnected Clinical Information Systems, (e.g., HIS, PACS);*
- *Mobile client devices, (e.g., mobile applications);*
- *Remote care system assets, (e.g., medical equipment for tele-monitoring);*
- *Data and records, (e.g., health records); *
- *Operating resources, (e.g. sterilization material).*

### Methodology

In the SAFECARE project the scenarios are divided into strategic and technical [8]. The strategic scenario describes key variables to recognize the *risk source*, *opponent/objective*, *stakeholder/intermediate events*, and *impact/severity*. The technical scenario describes a process of how an attacker can take control of some assets and cause incidents in health care organizations. The model for developing technical scenario consists of four phases:

1. *Know*: discover the vulnerabilities of health care organizations (ecosystem mapping, information on key people, and systems). These activities are directed by the attacker to prepare for his assault and increase his probabilities of success.
2. *Get in*: actions are taken by the attacker to introduce himself in the planned goal, either in cyber or physical assets (Hospital information System, Department rooms, PLC room, etc.).
3. *Find*: during this phase, once the attacker has access to health care assets physically or digitally, he identifies where are locate the desired material or data.
4. *Control*: In the last stage, the attacker takes control of the assets or resources (e.g., material, data). The consequence is sabotage, loss of patients’ data, ransomware.

In the SAFECARE project, 12 different Cyber-Physical technical scenarios were developed, involving the assets described. In the following of this paper we will consider a simplified scenario to show the interactions between the different modules of the platform.

### Scenario Description

Based on one of the incident scenario developed in SAFECARE, a sample scenario has been defined in order to test and show the functionalities of the system. In this scenario, an attacker gets the control of a medical device connected to the internal network in order to expose patients’ medical data. The main steps followed by the attacker are:

1. Obtain information of the medical device. Before going to the hospital, the attacker gets information on the medical devices, for example using Open Source INTelligence (OSINT) techniques, in order to identify their possible vulnerabilities.
2. Obtain local access to the medical device. The attacker goes to the area where the identified device is located.
3. Installation of malware on the medical device. The attacker install a malware software on the medical device through, for example, the connection of an USB flash drive. The malware propagates along the local network, in order to infect also the server with the medical records of the hospital patients, the final objective of the attack.
4. Exposition of medical data. When the malware infects the server with patients’ health record data, the attacker can access the data and expose them, with severe consequences on the reputation of the hospital as well as the possibility to modify them, putting in danger the patients’ life.

This scenario is a good example of integration between physical and cyber attacks, as in order to install the malware, the attacker must get the physical access to the area where the device is located and then wait until nobody else is present. BTMS is able to detect this suspicious loitering behaviour by analyzing live video streams and generates an event. Afterwards, two cyber anomalies are detected by CTMS: the installation of malicious software on the device and the infection of the server that contains patients’ data, generating two security events that are grouped by the operator in an cyber incident.

### Scenario Simulation

**Fig. 3.**
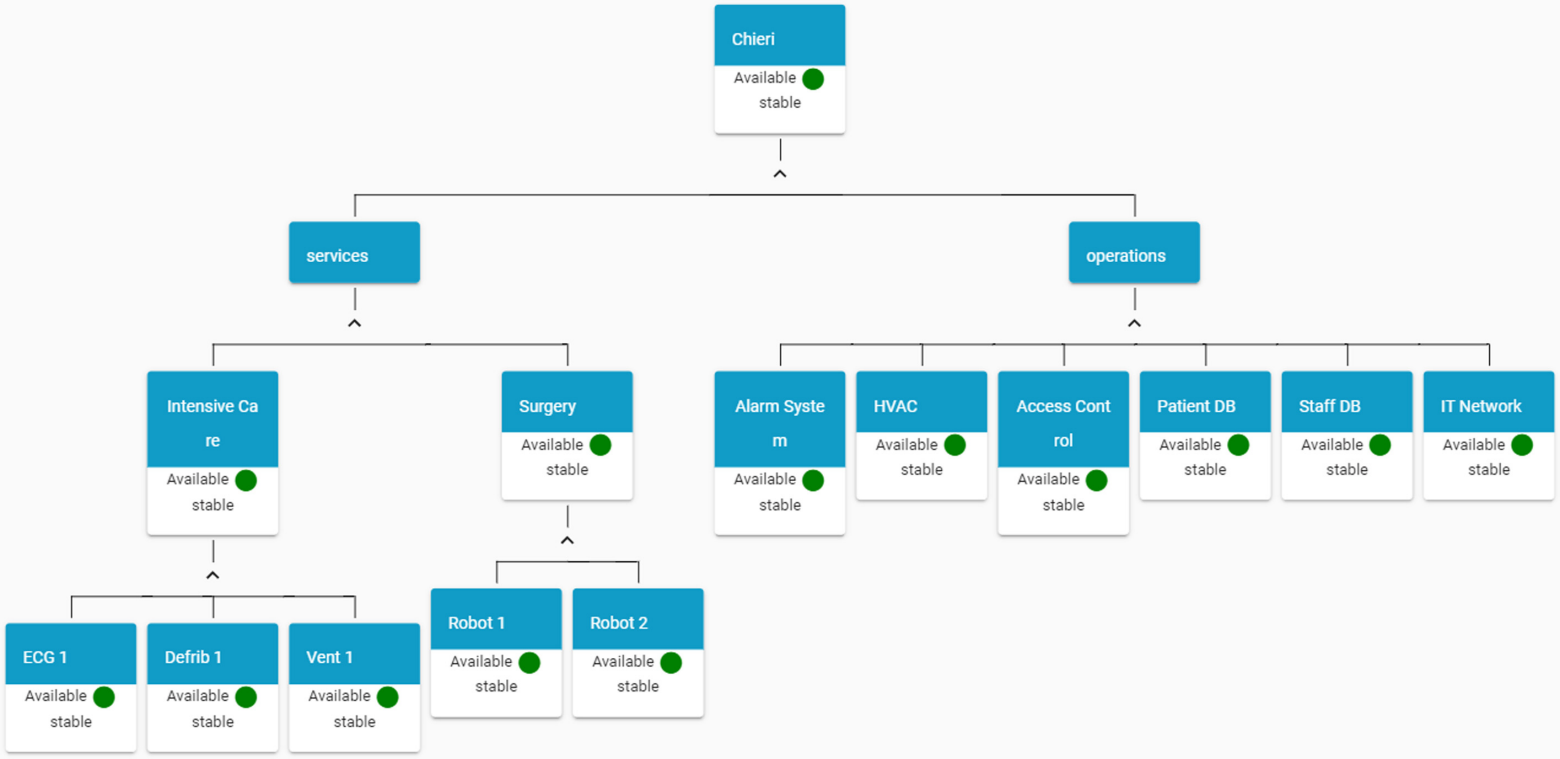
HAMS view at start-up (Color figure online)

In order to perform the simulation of a scenario, a sample hospital has been defined, and represented in Fig. 3.

This sample scenario firstly generates an incident message from the BTMS, detecting the loitering behaviour in a restricted area of the hospital. The main fields of the corresponding JSON message contain:
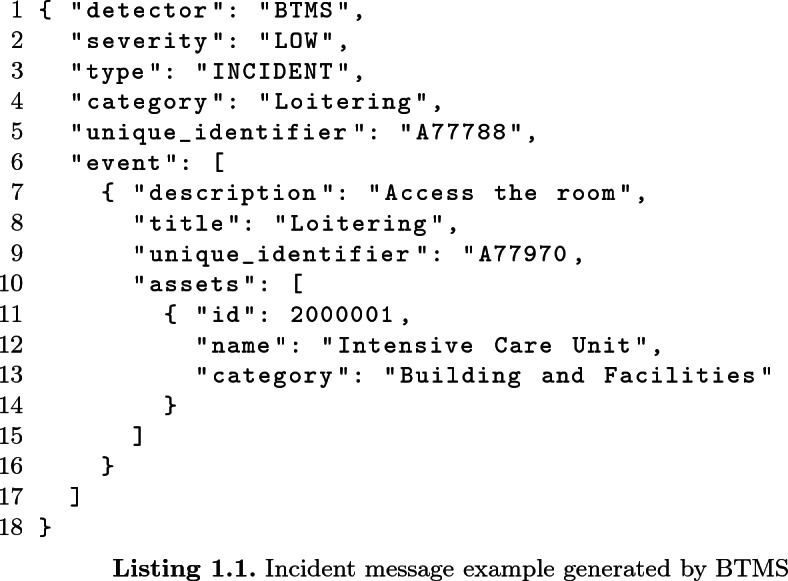


The *event* can include other parameters related to the equipment that initially detected the event as well as the links to media resources (videos records), that triggered the suspicious behaviour alert.

Upon receiving this incident message, the HAMS verify that the category of the incident is *Loitering*, with a severity level *LOW*. As a consequence, no actions are required on the assets, thus the status of the asset with id 2000001 (the “Intensive Care Unit” in the example) doesn’t change.

The next step of the attack is the infection of a medical device with malware in order to spread it over the network and expose patient health data. This cyber incident is detected by CTMS, the module responsible for detecting malicious cyber actions. The structure of the JSON message is similar to the previous one, but more events are considered. An extract of the resulting JSON is as follows:
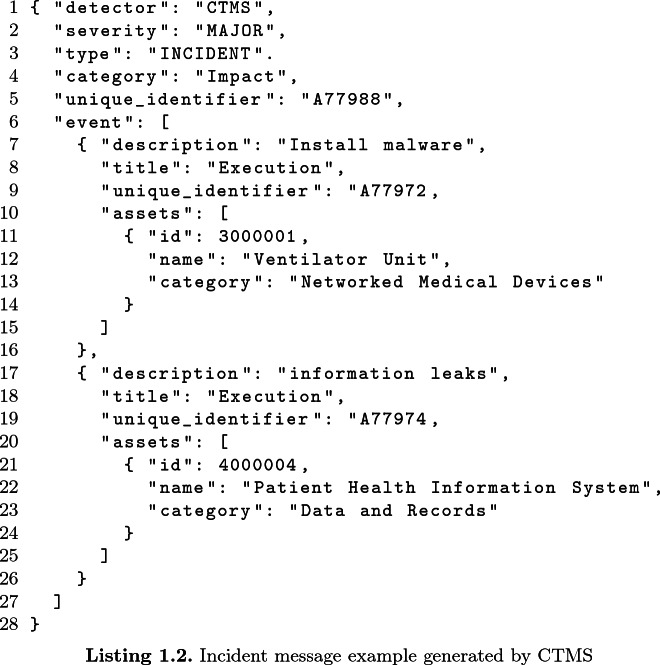


This message describes a major incident that involves two assets: a *Ventilator Unit* (with asset id 3000001) and the *Patient Health Information System* (with asset id 4000004). Both assets are heavily compromised and don’t work properly, thus their status must be changed to *unavailable*. According to the status description provided in Sect. 4, the Boolean value is set to *False*, the colour code is set to *red* and the stability is set to *deteriorating* (under the hypothesis that both assets were properly functions before the incident).

**Fig. 4.**
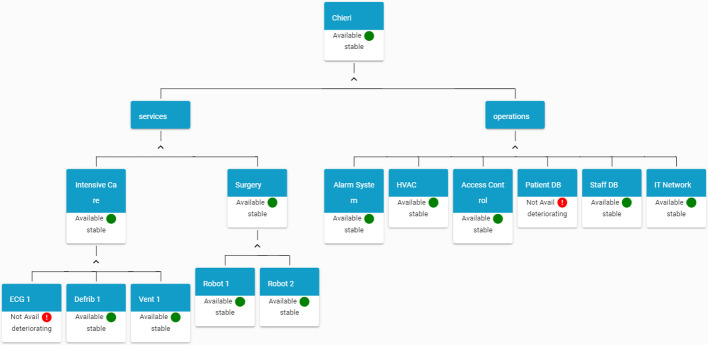
HAMS view after receiving an incident message (Color figure online)

Finally, the HAMS generates an *availability* message to make the other module of the system aware of the change of the availability status of the involved assets. The *availability* message is a JSON message that contains the *id* of the incident and the list of the assets with their new status. The structure of the message is as follows:
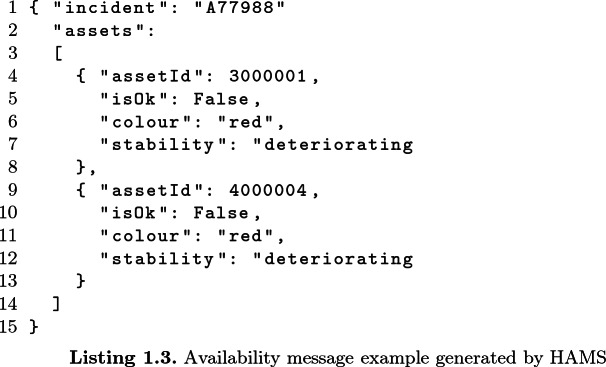


Figure 4 shows the HAMS graph after the elaboration of the incident message, where it is possible to see that the Ventilator Unit in the Intensive Care ward and the Patient DB service are not available anymore.

The cyber incident has a cascade effect also on other hospital assets. HAMS receives a further *impact message* generated by the IPDSM. This message contains a list of assets indirectly affected by the incident, each one associated with an *ImpactScore* parameter.

An example of *impact* message correlated with the *incident* message described above is as follows:
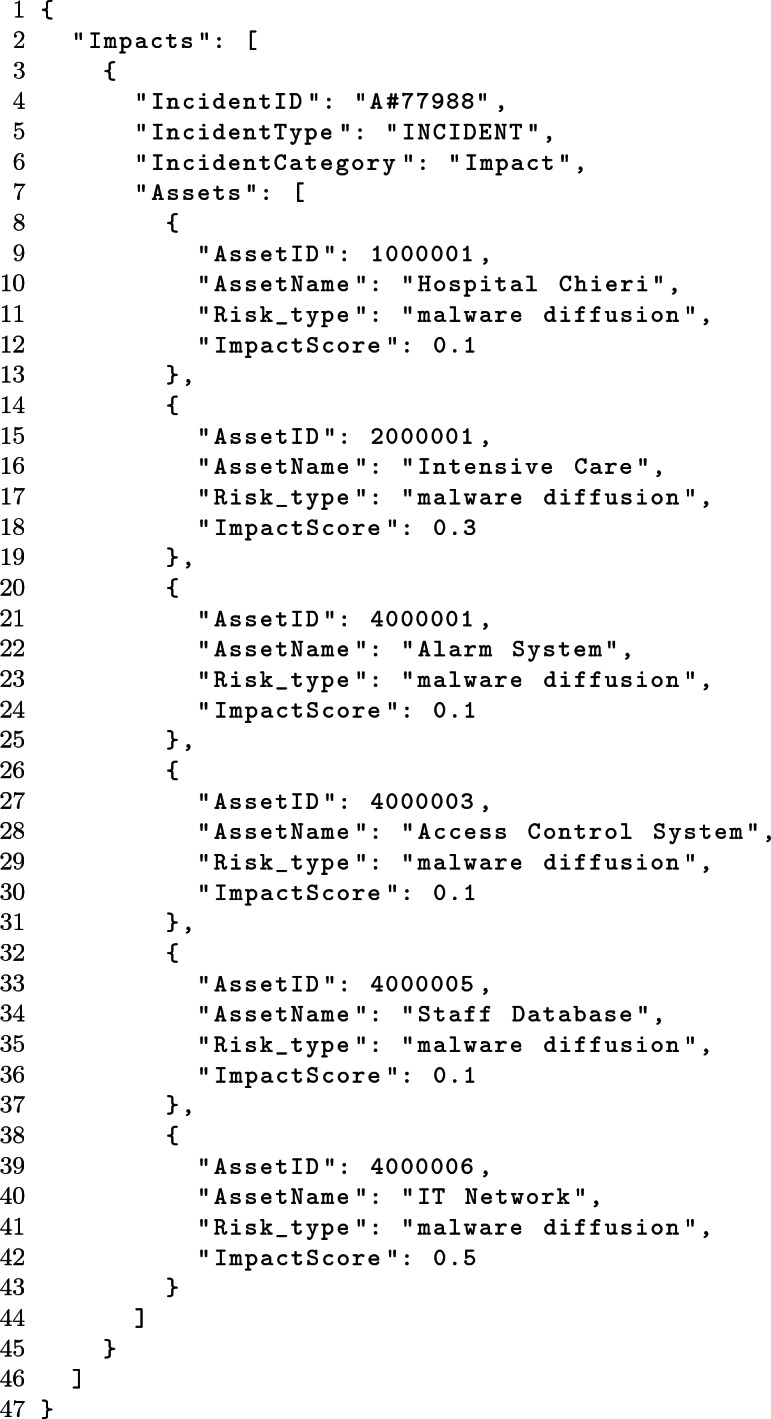

**Fig. 5.**
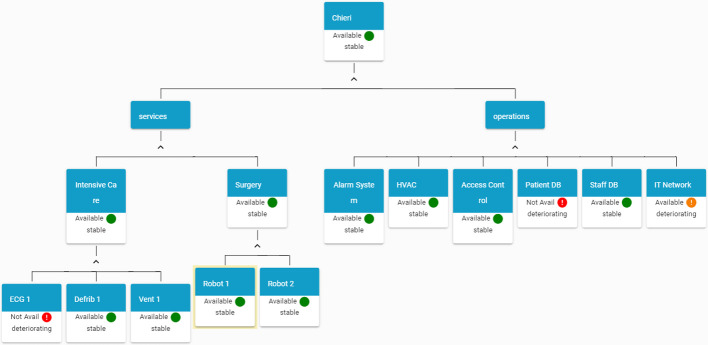
HAMS after receiving an impact message (Color figure online)

As it can be seen by the message, the *incident* has impacted various assets in the hospital. However, the majority of them reports an *ImpactScore* of 0.1, meaning that the impact is quite marginal and there is no need to change the status of the asset.

The spread of a malware software on the network compromises the normal operations of the IT Network service even if it is still working. A *yellow* status is the updated availability value.

Figure 5 shows the HAMS graph after the elaboration of the impact message. In the figure, it is possible to view that the status of *IT Network* becomes *yellow* as a cascade effect from the incident.

As at least one asset changed its availability status, a new *availability* message is generated, following the same structure of the one generated after the reception of the *incident* message.

## Conclusions and Future Work

The protection of Critical Infrastructures is a complex process that involves many pitfalls. SAFECARE project, and the modules presented in this paper, aim at enhancing the security and safety of healthcare and the *knowledge* about asset status of an hospital. This has potentially a great impact in term of benefits, especially during crisis situations like those experienced during COVID-19 peaks in the hardest hit areas, where specialized assets became quickly saturated and a percentage of patients needed to be transferred to other structures, in some cases even abroad.

The automated updating of assets status is an important feature in a context of evolving threats, where a single malware entering the internal communication networks can lead to harmful effects and fatal impacts on essential services for the people.

A further functionality that will be added to the platform will give the possibility of running a “demo mode”, simulating events and incidents without affecting the information stored in the database and trigger the other modules. This will be particularly useful to train the operators and design security exercises as a way of enhancing the awareness of all involved personnel.

## Footnotes

1 https://www.safecare-project.eu/.
